# The reliability and suitability of strength assessments in frail and pre-frail older adults: recommendations for strength testing in older populations

**DOI:** 10.1186/s12877-023-04552-3

**Published:** 2023-12-08

**Authors:** Bridgitte Swales, Gemma C. Ryde, Iain Fletcher, Anna C. Whittaker

**Affiliations:** 1 Faculty of Health Sciences and Sport, University of Stirling, Stirling, FK9 4LA Scotland UK; 2 The School of Cardiovascular and Metabolic Health, College of Medical, Veterinary and Life Sciences, University of Glasgow, Glasgow, Scotland, UK; 3 Institute for Sport and Physical Activity Research, University of Bedfordshire, Bedfordshire, England, UK

**Keywords:** Successful ageing, Frailty, Strength, Physical function, Reliability, Strength assessment

## Abstract

**Background:**

Lifelong strength is fundamental to physical function, health, and quality of life. Reliable appropriate strength assessment measures for older adults play an important role in effective evaluation of baseline ability and exercise prescription to counter disease and disuse. This study aimed to investigate the within-session reliability of maximal isometric knee extension and flexion, hip abduction and adduction, and handgrip strength measures in frail and pre-frail older adults.

**Method:**

The study was conducted at a residential care home in Birmingham, UK. All care home residents aged ≥ 65 years; pre-frail or frail according to the Fried Frailty phenotype criteria; able to speak and read English; not currently involved in any other clinical trial; without severe sensory impairments; and with a predicted life expectancy greater than the trial length were eligible. Maximal isometric lower limb testing was performed using specialised resistance training equipment and a portable measurement device, and grip strength was assessed using a portable dynamometer. All eligible participants attended a single testing session and performed three trials per measure. Peak force measures were obtained for analysis. Within-session reliability for each measure was calculated from repeated-measures analysis of variance, intraclass correlation coefficients (ICC), and coefficients of variation (CV) with 95% confidence intervals.

**Results:**

Eleven frail and eleven pre-frail older adults participated in the study. Within-session absolute and relative measures were found to be reliable with the highest overall repeatability indicated between trial 2 and trial 3 for knee extension, hip abduction, and handgrip (CV ≤ 4.65%, ICC ≥ 0.96) with variation evident across all measures, except knee extension, from trial 1 to 2.

**Conclusions:**

Overall, maximal isometric strength in frail and pre-frail older adults with no previous testing experience can be measured with good to high reliability within their first testing session. An initial two familiarisation trials followed by two measurement trials is recommended to achieve the highest level of overall repeatability.

**Trial registration:**

The trial was registered with ClinicalTrials.gov: NCT03141879 on 05/05/2017.

## Introduction

Muscle strength plays a critical role in health status throughout the life span. In concert with skeletal muscle mass, muscle strength offers both multisystemic and specific musculoskeletal benefits and underpins physical function and capacity [1, 2]. Age-related loss of muscle strength is strongly associated with an overall decrease in physical function [3], loss of independence [4] and adverse outcomes associated with frailty, falls and sarcopenia [5–7]. Frailty is a multicomponent clinically significant syndrome typified by reduced resistance to stressors and associated with an increased risk of falls, disability, and mortality [8].

Measuring muscle strength accurately and appropriately with reliable and easy to use devices is essential for case finding and diagnosis but also prevention and treatment strategies [7]. This is particularly important with mounting evidence supporting the role for resistance exercise in reversing or changing the trajectory of strength decline and frailty [9, 10]. Isometric measures of maximal strength are common in the published clinical and rehabilitation research [11, 12] and have been shown to be predictive of mortality [13], functional status and health outcomes [14] and clinically appropriate for older adults [15]. Isometric tests require minimal familiarity and movement skill [16]; are relatively easy to administer; pose minimal injury risk; and are less fatiguing than dynamic 1RM testing [17]. When compared to dynamic strength tests, this arguably makes them better suited for weaker and/or inexperienced participants [15]. Further, isometric tests can provide additional Rate of Force Development (RFD) data [16]. RFD has shown direct association with the ability to contract muscles rapidly and maximally, related to falls risk [1]. Handgrip strength is a commonly used isometric strength assessment in clinical, and research settings [18, 19]. Reasons include portability, simplicity, affordability, and ease of measurement [20, 21]. A recent review [22] concludes that handgrip strength has predictive validity for decline across mobility, functional status, cognition, and mortality, and it has been proposed as a biomarker of ageing. However, there is no universally agreed protocol for strength assessments with frail and pre-frail older adults [23].

Lower limb strength is frequently assessed in research, clinical and rehabilitation settings due to established relationships with Activities of Daily Living (ADL) [3], walking speed [24], and falls [25]. Further, lower limb measures may be more representative of functional ability and motor skills than grip strength [21, 26], emphasising that handgrip should not be relied on as a proxy for overall muscle strength as there is low to moderate agreement between measures of handgrip strength and knee extension force [27]. A combination of measures may provide a clearer indication of strength deficit [28, 29]. Previous research has focused on isometric knee extension test [12] due to its multiple clinical applications for older adults including screening, disability and falls assessment risk [30]. Other reported measures include knee flexion, hip abduction and hip adduction [31].

Established measurement devices of muscle strength include fixed laboratory or clinical based dynamometers, or portable hand-held dynamometers (HHD). Laboratory-based dynamometers are considered gold standard and have high levels of test re-test reliability [21]. However, time, cost and accessibility issues may limit practical application in a field setting. While lower reliability has been reported with HHD [32] this may be due to a lack of protocol standardisation and tester skill [21]. Improvements in reliability and practicality have been noted with the use of additional stabilisation [33]. Work with nursing home residents showed high relative and moderate absolute reliability of maximal isometric muscle strength measures for knee extensors and flexors, hip abductors and extensors, and elbow flexors and extensors Buckinx, Croisier [34]. Other studies have completed field-based assessments with a portable strain gauge [35] or used this in conjunction with resistance exercise training equipment [36, 37]. Data from healthy, active adults reported excellent test re-test reliability for peak knee flexion and extension using resistance exercise equipment and the Performance Recorder (HUR Ltd., Finland) [38]. However, to the authors’ knowledge, no study to date has examined the test re-test reliability of this methodology with knee extension, knee flexion, hip adduction and abduction measures with frail or pre-frail older adults in residential care. This thorough lower-limb analysis would bring insight to the suitability and reliability of these measures in assessment of health and help guide appropriate orientation and familiarisation for this participant group. Reliable testing protocols and equipment are required to ensure accurate evaluation and confidently detect meaningful changes in force production. Establishing within-session reliability and estimating measurement errors for muscle strength tests in frail and prefrail older adults is indispensable for accurate evaluation but has not yet been clearly defined. Consequently, this exploratory study aimed to (i) quantify the within-session reliability (repeatability) of lower limb isometric strength measures and handgrip strength in frail and pre-frail older adults within one session to establish ability prior to intervention and (ii) relate this to the feasibility and appropriateness of field-based strength testing measures with frail and pre-frail older adults.

## Method

### Participants

Participants were recruited by either a direct approach from a staff member, introduction to a member of the research team, or by voluntary attendance at a short introductory talk given by the Principal Investigator and researcher in the care home. Participants were screened against the following eligibility criteria: (a) resident in the care home; (b) age ≥ 65 years; (c) having at least three of the five Fried Frailty Phenotype Criteria (Adapted from Fried, Tangen [8]) for the frailty study, and one or two of the five Fried Frailty Phenotype Criteria (Adapted from Fried, Tangen [8]) for the pre-frailty study; (d) no severe sensory impairments that would profoundly impact upon their ability to participate; (e) ability to speak and read the English language; (f) not currently taking part in any other clinical trial which could potentially affect the results of this study; and (g) with a predicted life expectancy greater than the length of the trial.

Data collection took place between February 2019 and December 2019 at a residential care home in Birmingham, UK. Ethical approval was provided by London Harrow Research Ethics Committee. REC: 17/LO/1316. Protocol: RG_17-108 IRAS: 219616. The trial was registered with ClinicalTrials.gov: NCT03141879 on 05/05/2017.

### Design

This was a within-session reliability study. It was an analysis of a sub-set of data collected at baseline during randomised feasibility trials with frail [37] and pre-frail older adults (Swales et al., accepted 2023).

The full feasibility protocol has been published elsewhere [39] and amendments to the eligibility criteria and strength assessments have been documented [37] (Swales et al., accepted 2023). As both trials used the same methods, and were conducted by the same researcher, the data were combined to obtain a larger sample of older adults. Analysis of the reliability of the strength assessments has not been previously reported.

### Measures and equipment

#### Anthropometrics

Baseline measures of standing height (m) and body mass (kg) were recorded as documented in the full study protocol [39]. Height was recorded to the nearest 0.1 cm (Marsden HM-250P Leicester Portable Height Measure; Rotherham, UK) and body mass using scales to the nearest 0.1 kg (Marsden Chair Scales; Rotherham, UK).

#### Strength testing

Handgrip strength was assessed with a Takei grip strength dynamometer (T.K.K. 5401, Grip-D, Takei Scientific Instruments Co. Ltd, Tokyo, Japan) in an upright, seated position with the participants forearm resting on the chair arm. The wrist position was just over the end of the arm of the chair in a neutral position with thumb upwards, and feet flat on the floor. The researcher supported the weight of the dynamometer and gave verbal encouragement. Maximal voluntary isometric strength was reported in kg, and relative values per kilo body mass were also calculated, kg/kg.

Isometric maximal lower-limb strength testing was performed using premium line HUR SmartTouch resistance training equipment (4^th^ Generation; HUR Ltd., Kokkola, Finland) leg extension/curl (model 5530) and hip adduction/abduction (model 5520) machines, connected to Performance Recorder (PR) 9200 (HUR Ltd., Kokkola, Finland). The PR consists of a hand-held display unit and portable industrial grade strain gauge which attaches to a permanent bracket on the machine. Performance Recorder Software Suite 3.0 11.0 (HUR Ltd., Kokkola, Finland) was installed on the researcher’s laptop IBM ThinkPad X1 Laptop (Lenovo, China) and used to record all measurement data. All programme and equipment settings, test procedures and analysis were conducted according to methods detailed in the HUR Ltd. Performance Recorder Software Suite User Manual, 2010 (HUR Ltd., Kokkola, Finland) and HUR Isometric Measurement Instruction Guide, 2012 (HUR Ltd., Kokkola, Finland). All measurement angles were determined by machine sensor attachment sites and lever arm position reported as 120° for extension and 140° for flexion (with 180° = full extension) and 15° between legs for hip adduction/abduction (HUR Ltd., Kokkola, Finland).

Knee extension and flexion tests were completed in a seated, upright position with each participant’s back against the machine back-rest, and stabilisation straps secured across their body at the hip and the thigh of the tested leg prior to testing. Using the electrically adjustable back support and lever arm lengths, the near-seat roller was positioned under the knee joint to ensure the axis of rotation of the swing arm was aligned with the lateral epicondyle of the femur. The ankle pad was positioned on the front (for knee extension) or rear (for knee flexion) of the shank at a comfortable position proximal to the lateral malleolus. All seat and roller positions, and lever arm length were recorded in the programme software before testing. Participants performed bilateral hip adduction and abduction tests in a seated, upright position with their back supported by the machine back-rest and each leg in an individual, padded support bracket. The brackets were non-adjustable, and depending on participant lower limb length, provided support behind the knee and shank.

### Procedure

Individuals completed all the measures on-site and at two separate testing sessions, separated by at least one week. In session one, participants completed the handgrip (HG) strength test as part of eligibility screening. In session two, participants performed unilateral knee extension and flexion tests, and bilateral hip adduction and abduction measures. The full study timeline is detailed previously [37].

No specific warm-up was completed before session one. Following one practice trial, the HG strength test was performed three times using the dominant hand, with 60s between trials. All participants completed a standardized warm-up before session two. This comprised two sets of 12 repetitions at light-moderate intensity (Rating of Perceived Exertion, (RPE) 3–5) with 60s recovery between sets and was performed bilaterally on all test machines (leg extension/curl, and hip abduction/adduction).

Following sensor attachment, participants performed one practice trial on the HUR machines. After a 60s recovery, participants completed three trials of five seconds with a minimum rest of 60s between trials. Each trial was initiated by a “3,2,1 Go” countdown with corresponding audible beeps from the software, and verbal encouragement. All three trials were completed on each measure before re-positioning for the next test. All machine-based measures were taken in the same order (left knee extension, left knee flexion, right knee extension, right knee flexion, adduction, abduction) and reported using Performance Recorder software (HUR Ltd., Kokkola, Finland). Absolute maximal voluntary isometric strength was reported as peak torque (Nm), and relative values were reported as peak torque divided by the participants body mass in kg, (Nm/kg).

### Statistical analysis

Initially all HUR force data were exported into Microsoft Excel™ and combined with measures of grip strength recorded in individual case report forms. All data was later transferred into IBM® SPSS® Statistics Version 28.0 for further analysis. Data cleaning was performed and included screening descriptive data for cases of statistical outliers, errors, erroneous inliers, and other extreme values. After identification, any suspected cases were checked against original case report forms and excluded from analysis if there was documented protocol violation or technical error. Separate analysis was performed with and without excluded data points. Descriptive statistics (means ± standard deviations) were calculated for all force variables for the whole group, men, and women. The assumption of normality was assessed via the Shapiro–Wilk test. A repeated-measures analysis of variance (ANOVA) was conducted to establish reliability within sessions (trials 1, 2 and 3) on each strength measure. Statistical significance was set at an alpha level of *p* < 0.05. Sphericity was assessed via Mauchley’s Test, and where violated, Greenhouse–Geisser was applied. A Bonferroni post-hoc test was used to identify pairwise differences. Within-session test–retest reliability was determined using coefficient of variation (CV) and intraclass correlation coefficient (ICC) to establish both absolute and relative reliability. Based on prior recommendation [40] ICC [1, 3] a two-way mixed effects model with absolute agreement was calculated. ICC values were classified where scores < 0.5 poor, 0.5–0.75 moderate, 0.75–0.9 good, and > 0.9 excellent. The level of reliability was based on the 95% confidence interval, not the ICC estimate itself [40]. As regards to CV%, acceptable thresholds were determined as < 10%. Overall repeatability was classified as very high (CV ≤ 5%, ICC ≥ 0.95), high (CV ≤ 10%, ICC ≥ 0.90) and moderate (CV ≤ 15%, ICC ≥ 0.80), in accordance with previous reliability studies [41] including intra-session repeatability studies of maximal isometric lower limb testing in older adults in care homes [35]. The reliability sections of this study are described based on the guidelines for reporting reliability and agreement studies [42]. As this was a feasibility study, an a priori sample size calculation was not performed.

## Results

### Participant and within-session descriptive statistics

Twenty-two older adults (*n* = 11 frail, *n* = 11 pre-frail) with a mean age of 83.4 (SD = 6.37) years ranging from 73 to 95 (13 female) were included. Frailty status was determined using the Fried frailty phenotype criteria [8]. Participants reported no injuries at the time of testing, no previous experience of resistance training or the isometric strength testing procedures. Baseline participant characteristics are shown in Table 1.

**Table 1:** Participant characteristics

Variable	Mean (SD)/n (%)
Age (years)	83.4 (6.37)
Age Range (years)	73–95
Gender—Female	13 (59.0)
Height (m)	1.62 (0.09)
Body Mass (kg)	74.1 (16.58)
Body Mass Index (BMI) (kg/m^2^)	28.2 (4.43)
Medical conditions	2.1 (1.4)
Fried frailty score (0–5)	2.3 (1.1)
Pre-frail (0–2)	11 (50.0)
Frail (3–5)	11 (50.0)
Fried Frailty criteria met	
Unintentional weight loss	1 (4.5)
Self-reported exhaustion	9 (40.9)
Weakness (grip strength)	15 (68.2)
Slow walking speed	10 (45.5)
Low physical activity level	18 (81.8)

Fried frailty score is calculated using Fried Frailty Phenotype criteria. The Fried frailty phenotype proposes that frailty be defined as a clinical syndrome in which 3 or more of the five following criteria are present, and pre-frailty in which 1 or 2 criteria are present: unintentional weight loss (> 10lbs in the past year), self- reported exhaustion, weakness (grip strength), slow walking speed, and low levels of physical activity [8]

Repeated measures ANOVAs identified variation in most measures across trials 1–3, with a general pattern of increase in mean score across all measures identified, except for left and right knee extension. Full descriptive statistics and results of ANOVAs are shown in Table 2.

**Table 2:** Means, standard deviations, and analysis of variance for all strength measures

Measure		Trial 1		Trial 2		Trial 3		ANOVA
	*n*	M	SD	M	SD	M	SD	
Absolute Peak Torque (N·m)								
L Knee Extension	20	66.2_a_	32.24	70.1_a_	31.67	68.6_a_	29.02	F (1.4, 26.7) = 2.33, *p* = .13, *n*^*2*^ = .11
R Knee Extension	20	72.7_a_	26.03	74.4 _a_	26.97	75.7 _a_	29.13	F (3, 38) = 1.95, *p* = .16, *n*^*2*^ = .09
L Knee Flexion	22	31.9_b_	13.05	33.3_b_	13.30	35.1 _a_	12.39	F (1.5, 30.8) = 5.26, *p* = .02, *n*^*2*^ = .20
R Knee Flexion	21	36.3_b_	15.41	39.2_ab_	16.20	40.6_a_	18.47	F (2, 40) = 5.19, *p* = .01, *n*^*2*^ = .21
Adduction	22	90.4_b_	29.97	92.2_b_	30.23	100.3_a_	34.59	F (2, 42) = 9.37, *p* = < .001, *n*^*2*^ = .31
Abduction	19	67.5_a_	24.85	70.8 _ab_	26.00	71.8_b_	25.60	F (2, 36) = 6.45, *p* = .004, *n*^*2*^ = .26
Absolute Peak Force (kg)								
Handgrip	22	21.9_a_	8.01	23.1_b_	8.07	23.2_b_	8.42	F (1.6, 32.8) = 7.88, *p* = .003, *n*^*2*^ = .27
Relative Peak Torque (N·m/kg)								
L Knee Extension	20	0.89_a_	0.34	0.95_a_	0.34	0.93_a_	0.32	F (1.5, 27.8) = 3.04, *p* = .08, *n*^*2*^ = .14
R Knee Extension	20	0.97_a_	0.30	0.99_a_	0.32	1.01_a_	0.34	F (2, 38) = 3.04, *p* = .08, *n*^*2*^ = .10
L Knee Flexion	22	0.43_b_	0.16	0.45_b_	0.17	0.48_a_	0.16	F (1.5, 32.3) = 7.03, *p* = .01, *n*^*2*^ = .25
R Knee Flexion	21	0.48_b_	0.16	0.51_ab_	0.16	0.53_a_	0.18	F (2, 40) = 4.62, *p* = .02, *n*^*2*^ = .19
Adduction	22	1.22_b_	0.31	1.25_b_	0.34	1.35_a_	0.34	F (2, 42) = 12.42, *p* = < .001, *n*^*2*^ = .37
Abduction	19	0.94_b_	0.23	0.98_ab_	0.25	0.99_a_	0.24	F (2, 36) = 5.23, *p* = .01, *n*^*2*^ = .23
Relative Peak Force (kg/kg)								
Handgrip	22	0.30_b_	0.08	0.31_a_	0.08	0.31_a_	0.08	F (1.5, 31.8) = 6.74, *p* = .01, *n*^*2*^ = .24

Means with different subscripts (not sharing any letter) indicate pairs of means which differ significantly at α = .05 level as indicated by Bonferroni procedure. a = means not significantly different from other means marked a or including a; b = means not significantly different from other means marked b or including b

### Absolute reliability: coefficient of variation (CV)

Across absolute and relative comparisons, no differences emerged between tests or limb tested, so the results are narratively summarised for both below. Pairwise intra-session comparisons found that CV ranged from 6.26% to 12.01% between trial 1 (T_1_) and trial 3 (T_3_). All measures, except left and right knee flexion, were < 10% indicating high reliability. Pairwise comparisons between T_1_ and trial 2 (T_2_) revealed CV of < 10% across all measures ranging from 4.73% to 9.97%. Notably hip adduction and handgrip measures were < 5% suggesting very high reliability. CV ranged from 3.40% to 8.31% between T_2_ and T_3_ indicating high to very high reliability across all measures: very high values of < 5% were found for knee extension, abduction, and handgrip. CVs and ICCs across all pairs of trials are shown in Table 3.

**Table 3:** Within-session reliability comparison for all strength measures across three trials

Measure	Trial 1–3	Trial 1–2	Trial 2–3	Trial 1–3	Trial 1–2	Trial 2–3
	CV [95% CI]	CV [95% CI]	CV [95% CI]	ICC [95% CI]	ICC [95% CI]	ICC [95% CI]
Absolute Peak Torque (N·m)						
L Knee Extension	8.50 [5.41, 11.59]	7.48 [4.59, 0.37]	4.49 [2.35, 6.62]	0.94 [0.87, 0.98]	0.97 [0.90, 0.99]	0.98 [0.95, 0.99]
R Knee Extension	6.26 [3.70, 8.82]	5.59 [3.20, 7.98]	4.65 [3.18, 6.11]	0.95 [0.88, 0.98]	0.97 [0.93, 0.99]	0.98 [0.95, 0.99]
L Knee Flexion	12.01 [8.22, 15.80]	7.81 [5.13, 0.49]	8.31 [4.99, 11.63]	0.88 [0.67, 0.95]	0.93 [0.84, 0.97]	0.96 [0.89, 0.99]
R Knee Flexion	10.91 [7.79, 14.03]	9.97 [6.65, 3.30]	7.17 [3.54, 10.80]	0.90 [0.69, 0.97]	0.92 [0.79, 0.97]	0.93 [0.84, 0.97]
Adduction	7.34 [4.13, 10.55]	4.96 [2.75, 7.18]	5.85 [3.01, 8.69]	0.88 [0.57, 0.96]	0.96 [0.91, 0.98]	0.91 [0.69, 0.97]
Abduction	7.57 [4.45, 10.68]	5.85 [2.51, 9.19]	3.40 [1.98, 4.83]	0.96 [0.81, 0.99]	0.98 [0.93, 0.99]	0.99 [0.97, 1.00]
Absolute Peak Force (kg)						
Handgrip	6.71 [4.17, 9.25]	4.73 [2.86, 6.59]	3.94 [2.57, 5.31]	0.96 [0.86, 0.99]	0.98 [0.87, 0.99]	0.98 [0.96, 0.99]
Relative Peak Torque (N·m/kg)						
L Knee Extension	8.50 [5.41, 11.59]	7.48 [4.59, 0.37]	4.49 [2.35, 6.62]	0.92 [0.81, 0.97]	0.95 [0.84, 0.98]	0.98 [0.95, 0.99]
R Knee Extension	6.26 [3.70, 8.82]	5.59 [3.20, 7.98]	4.65 [3.18, 6.11]	0.94 [0.84, 0.97]	0.95 [0.89, 0.98]	0.97 [0.93, 0.99]
L Knee Flexion	12.01 [8.22, 15.80]	7.81 [5.13, 0.49]	8.31 [4.99, 11.63]	0.87 [0.61, 0.95]	0.94 [0.85, 0.97]	0.95 [0.85, 0.98]
R Knee Flexion	10.91 [7.79, 14.03]	9.97 [6.65, 3.30]	7.17 [3.54, 10.80]	0.85 [0.58, 0.94]	0.87 [0.69, 0.95]	0.89 [0.75, 0.95]
Adduction	7.34 [4.13, 10.55]	4.96 [2.75, 7.18]	5.85 [3.01, 8.69]	0.84 [0.38, 0.95]	0.95 [0.88, 0.98]	0.89 [0.58, 0.96]
Abduction	7.57 [4.45, 10.68]	5.85 [2.51, 9.19]	3.40 [1.98, 4.83]	0.91 [0.67, 0.97]	0.93 [0.81, 0.97]	0.97 [0.92, 0.99]
Relative Peak Force (kg/kg)						
Handgrip	6.71 [4.17, 9.25]	4.73 [2.86, 6.59]	3.94 [2.57, 5.31]	0.92 [0.76, 0.97]	0.96 [0.77, 0.99]	0.96 [0.92, 0.99]

*L* left, *R* right

### Relative reliability: intraclass correlation coefficient (ICC)

Pairwise comparisons for T_1_ and T_3_ using absolute values reported ICC values ranging from 0.88 to 0.96 with large confidence intervals (CI) across some measures. Notably, adduction and knee flexion (R and L) measures were classified as ‘moderate to excellent’ reliability, with all other measures classed as ‘good to excellent’, using CI as the basis for evaluation. Pairwise comparisons between T_1_ and T_2_ revealed ICC values for all measures ranging from 0.92–0.98 (95% CI = 0.79, 0.99) with right knee extension, adduction and abduction values representing ‘excellent’ reliability. ICC values ranged from 0.91 to 0.99 (95% CI = 0.69, 1.00) between T_2_ and T_3_, with knee extension measures (right and left), abduction and handgrip rated as ‘excellent’.

Pairwise comparisons for T_1_ and T_3_ (relative) revealed ICC values ranging from 0.84 to 0.94 with large confidence intervals across adduction and knee flexion measures. Adduction test reliability ranked ‘poor’ to ‘excellent’ whereas all other measures classified either ‘moderate’ to ‘excellent’ (knee flexion and hip abduction) or ‘good’ to ‘excellent’ (knee extension and handgrip) [40]. Pairwise comparisons between T_1_ and T_2_ revealed ICC values for all measures ranging from 0.87–0.96 (95% CI = 0.69, 0.99), and rating ‘good’ to ‘excellent’ apart from right knee flexion, which was classified ‘moderate to excellent’. ICC values ranged from 0.89 to 0.98 (95% CI = 0.58, 0.99) between T_2_ and T_3_ for all measures, with knee extension (right and left), abduction and handgrip rated as ‘excellent’.

### Overall repeatability

All pairwise measures, except adduction, achieved progressively higher overall repeatability across trial comparisons indicating improved test re-test reliability: T_2_ to T_3_ > T_1_ to T_2_ > T_1_ to T_3_. In absolute and relative terms, T_2_ to T_3_, hip abduction, knee extension, and handgrip measures report ‘very high’ overall repeatability (CV ≤ 5%, ICC ≥ 0.95), with right knee flexion classified as ‘high’ (CV = 7.17%, ICC = 0.93 (absolute) and 0.89 (relative). Adduction and knee flexion measures both displayed ‘high’ and ‘moderate’ overall repeatability, in absolute and relative terms, respectively.

### Feasibility and appropriateness

Being able to complete the measures above reliably and accurately without risk of injury showed that these field-based strength testing measures were feasible and appropriate for use with frail and pre-frail older adults.

## Discussion

The aims of this study were to (i) quantify the within-session reliability of lower limb isometric strength measures and hand-grip strength in frail and pre-frail older adults and (ii) relate this to the feasibility and appropriateness of field-based strength testing measures with frail and pre-frail older adults. The main study finding indicates that isometric hand grip and lower limb strength can be assessed in a field-based setting with the specialised equipment used in this study with high reliability in frail and pre-frail older adults. The results confirm previous findings that isometric strength can be reliably evaluated using a portable measurement device and specialised gym equipment. The findings also show that lower limb strength in frail and pre-frail older adults with no previous testing experience can be measured with good to high reliability within the first testing session. Overall, the results suggest high levels of within-session reliability across all measures with highest overall repeatability indicated between T_2_ and T_3_ and for knee extension, hip abduction, and handgrip strength. This suggests that two practice familiarisation trials and two data collection trials would be reliable in this setting with the specialised equipment used in this study, but more practices and repeats may yield slightly higher reliability.

Previous studies and current testing guidelines agree with the present findings in frail and pre-frail participants by underscoring the importance of an appropriate warm-up and familiarisation process prior to isometric strength testing [43] and indicate that an initial practice trial and at least a further two trials are necessary to obtain an accurate maximal strength value [44]. Research suggests that the reliability of a strength test may develop with repetition and be influenced by unfamiliar or non-practised conditions [45] which is supported by the present finding of better reliability between trials 2 and 3 rather than the first trial and subsequent trials. It has been suggested that older adults, particularly those unaccustomed to strength training or testing, may additionally require more practice and familiarisation [46, 47]. In agreement with this, the present research study identified variation across trials, with a general pattern of increase in mean scores across all measures across the three trials after the initial practice trial. This is likely to be related to a ‘learning effect’ between trials [48] and could potentially be attributed to the omission of a separate familiarisation session [49]. However, given the practical implications of additional sessions for this population group including time constraints, costs, and increased participant burden, additional extra familiarisation sessions may not be feasible. The present data suggest two practice trials then two actual trials would be appropriate for high repeatability in future investigations in a single-session test protocol.

There are equipment differences between the present study and previous research, precluding absolute direct comparison. However, CV for maximal isometric grip strength was rated good to high across all trial comparisons and indicated the highest levels (very high ≤ 5%) of reliability across all measures between T_2_ and T_3_, (CV = 3.94%). These findings compare favourably with previous studies which reported CV for maximal voluntary isometric grip strength in older men as 10.93% [50] and 5.18–7.63% [51] in community dwelling older adults.

According to the present findings, all lower limb measures, between T_2_ and T_3_, can be assessed with high to very high reliability (CV = 3.40 – 8.31%) with higher levels of reliability indicated in knee extension and abduction measures (CV < 5%). These results are in line with reported findings of CV ≤ 6.0% for intra-session repeatability in isometric knee extension tests with institutionalised older adults [35] and CV = 3.0% (range 0–6.0%) in older women [52]. The current study found CV for hip abduction of 3.40% indicating high reliability. To date, the research on hip abduction measures utilises a variety of different protocols, positions, and equipment, so there is limited direct comparison. However, the findings do corroborate earlier work [53] which found that hip abductor strength could be measured reliably in older adults in varying positions. Hip abductor strength has been shown to have good diagnostic accuracy to distinguish between fallers and non-fallers, and future studies should focus on the evaluation of reliable, field-based testing solutions for older adults [54].

It is interesting to note that reliability for knee flexion measures in this study was less consistent than other reported measures with large confidence intervals, showed notable differences between right and left leg reliability, and differed from previous research findings [52]. Possible explanations for this could be the small sample size, unfamiliar movement pattern and unilateral action [43] or protocol differences with other studies which identified the participants dominant and non-dominant limb [38, 41]. Limb dominance was not recorded as part of the current study and may be a useful consideration for further research. However, the present study suggests that knee flexion may be less valuable in comparison to knee extension, particularly when time is limited and/or participant burden is high, particularly in vulnerable participants such as older frail and pre-frail adults.

Relative reliability was good across most measures for absolute and relative values with the highest levels of reliability consistently reported for handgrip, knee extension and abduction, ICC ≥ 0.96. High levels of reliability for knee extension peak torque matched those observed in earlier studies using a laboratory-based dynamometer which reported within session reliability (ICC 0.99–1.00) with older women [52]. The current study findings were similarly found by others [15, 53] who reported high levels of reliability, feasibility, and clinical relevance for maximal voluntary isometric strength testing for hip abduction in standing and supine positions in older adults. Others [54] also reported good levels of reliability for hip adduction although it is important to interpret direct comparisons with caution due to differences in equipment, protocol, and positioning. Even so, the present findings suggest that hip abduction measures may be more valuable than adduction measures if time limits using both.

Handgrip is consistently used as a strength measure, not least due to its relative low cost and portability, and the present data in frail and pre-frail older adults contribute further confirmation that reliability is high in this population. However, while handgrip strength measures may be considered a proxy for global strength, there is growing recognition that a combined assessment including measures of isometric lower limb strength, as noted in this study, may offer a more comprehensive evaluation.

With regards to the testing equipment, previous reliability trials that used HUR specialised gym equipment (HUR Ltd., Finland) and a portable measurement device [38] reported excellent test re-test reliability for knee extension and knee flexion measures with healthy adults on their dominant leg: peak knee flexion torque (ICC = 0.96 (95% CI: 0.85, 0.99)) and peak knee extension torque (ICC = 0.96 (95% CI: 0.87, 0.99)). It is encouraging to compare the current study findings and note corresponding high levels of reliability across right and left leg measures of peak knee flexion (ICC = 0.93 – 0.96 (95% CI: 0.84, 0.99)) and for both limbs with peak knee extension (ICC = 0.98 (95% CI: 0.95 – 0.99)). However, the large confidence intervals for peak knee flexion, reported in relative terms, suggest that these should be viewed with some caution. Further, the present findings are specific to older adults with pre-frailty or frailty so this adds data in a novel population to the current literature but also suggests that with this population, knee extension may be a preferable measure to flexion.

### Limitations

The sample size is small in relation to the aims of the study. Additionally, the scope of the study did not extend to comparisons across conditions with different numbers of practice tests or actual trials. However, it did offer valuable insight into the practical implications for future strength testing, concluding that two practice trials and two actual trials offer the highest level of repeatability. It is a strength of the present study that it demonstrates that strength testing is feasible in older adults and gives clear recommendations for the number of practice tests and trials optimal for reliability and repeatability.

The present study utilised four specific lower limb tests and three trials per test, but for some participants and contexts, this may be too much. However, the study has shown that measures of knee extension, hip abduction and handgrip strength may be preferable if time is limited, and participant burden is a concern.

Finally, it is important to note that the specialised equipment used for lower limb testing in this study may not be accessible or financially viable for all residential care facilities and has limited portability for researchers in relation to field testing. This limits practicality and generalisability in many residential care settings. In which case further data supporting the reliability of handgrip strength from this study can at least inform practitioners that they are using an appropriate strength assessment tool which is also more affordable and portable.

### Recommendations and future directions

The development of practical and reliable field test measures for maximal isometric strength is challenging, and particularly for frail older adults in residential care facilities. In terms of the present study, measures of knee extension, hip abduction, and handgrip strength are identified as the best options. Although there is variation across trials, the data also support the use of two practice trials and two real trials for high reliability.

The specialised resistance training equipment used in this study provides a reliable measure of maximal isometric strength in frail older adults that could be used in a clinical, research or rehabilitation setting. Issues with practicality, generalisability and economic viability may limit wider use in residential care facilities and would need further consideration. However, data regarding the reliability of handgrip strength from this study, lends further support to its use as an appropriate assessment tool which is also more affordable and portable.

Finally, given the limited capacity of this study to test a range of conditions with different numbers of practice trials and actual trials, future research in an experimental setting may be valuable to determine the optimal number of each. However, the present study does strongly support the ‘two-plus-two’ design which may be more feasible and practical than longer protocols which may only provide incremental improvements in reliability e.g., from high to very high across all measures.

## Conclusion

This study shows the appropriateness of isometric hand grip and lower limb strength measures, using the specialised equipment in this study, in a field-based setting with high within-session reliability in frail and pre-frail older adults with no testing experience in their first testing session. For optimal repeatability in a manageable protocol design, we would recommend, where possible, testing knee extension, hip abduction, and handgrip strength with two practice trials followed by two measurement trials. A larger-scale study in this population would confirm the reliability further.

## Data Availability

The datasets generated and/or analysed during the current study are not publicly available due to still being used for further analysis and PhD thesis work but will be published upon acceptance of the final manuscript and PhD completion associated with these data but are available from the corresponding author on reasonable request.

## Abbreviations

ADL: Activities of daily living
ANOVA: Analysis of variance
BMI: Body mass index
CI: Confidence interval
CV: Coefficient of variation
HG: Handgrip
HHD: Hand-held dynamometer
ICC: Intraclass correlation coefficient
PR: Performance Recorder
RPE: Rating of Perceived Exertion
SD: Standard deviation
